# Supplementary material for: Exploring the multilevel determinants of low birth weight in Bangladesh: Understanding implications for targeted public health interventions
Source: PLOS Glob Public Health. 2026 Jan 23;6(1):e0005823. doi: 10.1371/journal.pgph.0005823 (PMC12829852; doi:10.1371/journal.pgph.0005823)
Supplement: S1 Table — (DOCX) [file pgph.0005823.s001.docx]

**S1 Table.** Variables included in the study and their coding for analysis.

| Variable Names | Type | Descriptions | Categorization |
| --- | --- | --- | --- |
| Dependent Variable | | | |
| Low Birth Weight | Nominal | Low birth weight <2500 gm; Normal birth weight ≥ 2500 gm | 1=Low birth weight; 0=Normal birth weight. |
| Independent Variables | | | |
| Division | Nominal | The administrative area where the respondents live. | 1=Barishal; 2=Chattogram; 3=Dhaka; 4=Khulna; 5=Mymensingh; 6=Rajshahi; 7=Rangpur; and 8=Sylhet. |
| Mother’s age | Numeric | Mother’s age in years & Range 15-49 | 1=15-19; 2=20-24; 3=25-29; 4=30-34; 5=35-39; 6=40-44; 7=45-49. |
| Mother education | Ordinal | Mother’s education Level | 1=No education; 2=Primary; 3=Secondary; 4=Higher. |
| Wealth Index | Ordinal | Classification of respondents based on the wealth index. | 1=Poorer; 2=Poor; 3=Middle; 4=Rich; and 5=Richer. |
| Type of place | Nominal | Type of place of residence | 0=Urban; and 1=Rural. |
| Mother’s work | Nominal | Respondent currently working | 0=No; and 1=Yes. |
| Child’s sex | Nominal | Sex of the child. | 0=Male; and 1=Female. |
| Child is alive | Nominal | The child is alive | 0=No; and 1=Yes. |
| Cesarean delivery | Nominal | Delivery by cesarean section. | 0=No; and 1=Yes. |
| Parity | Nominal | Total children ever born | 1=less than 5; 2= greater than or equal 5. |
| Iron tablets/syrup | Nominal | Taking iron tablets/syrup | 0=No; 1=Yes; and 2=Don’t know. |
| Child is twin | Nominal | Single or multiple births | 0=Single birth; 1=1^st^ of multiple; 2=2^nd^ of multiple. |
| Preceding birth interval | Numeric | 1=Very short, 2=Short interval, 3=Optimal interval, 4=Long interval | 1=<18 months; 2=18-23 months; 3=23-59 months;4=$\geq60$months |
| Decide healthcare | Nominal | Person who usually decides on: respondent's health care. | 1=respondent alone, 2=respondent and husband/partner, 3=husband/partner alone, 4=someone else, and 5=other. |
| Child birth size | Nominal | Size of child at birth | 1=Very large, 2=Larger than average, 3=Average, 4=Smaller than average, 5=Very small, and 6=Don’t know. |
| ANC visits | Nominal | No. of antenatal visits during pregnancy | 0=≤ 2 visits; 1=> 2 visits |
| Delivery place | Nominal | Place of delivery | 0=Home; 1=Hospital; 2=Other. |
| BMI (kg/m^2^) | Nominal | Body mass index (BMI) | 1=≤18.5; 2=18.5-24.9; 3=25.0-29.9 |
