# Supplementary material for: Exploring the multilevel determinants of low birth weight in Bangladesh: Understanding implications for targeted public health interventions
Source: PLOS Glob Public Health. 2026 Jan 23;6(1):e0005823. doi: 10.1371/journal.pgph.0005823 (PMC12829852; doi:10.1371/journal.pgph.0005823)
Supplement: S2 Table — (DOCX) [file pgph.0005823.s002.docx]

**S2 Table.** Logistic regression model for examining the relationship between Low Birth Weight (LBW) and others factors for unadjusted odds ratio.

| **Factors** | **Category** | **Unadjusted OR (95% C.I.)** | **P-value** |
| --- | --- | --- | --- |
| Division | Dhaka | Reference | |
|  | Barishal | 0.69 (0.45, 1.05) | 0.084 |
|  | Chattogram | 0.72 (0.50, 1.04) | 0.078 |
|  | Khulna | 0.56 (0.37, 0.85) | <0.01*** |
|  | Mymensingh | 0.40 (0.26, 0.60) | <0.01*** |
|  | Rajshahi | 0.45 (0.30, 0.70) | <0.01*** |
|  | Rangpur | 0.55 (0.36, 0.85) | <0.01*** |
|  | Sylhet | 0.66 (0.43, 0.99) | <0.05** |
| Residence type | Urban | Reference | |
|  | Rural | 0.73 (0.56, 0.95) | <0.05** |
| Education level | No education | Reference | |
|  | Primary | 0.77 (0.46, 1.29) | 0.320 |
|  | Secondary | 1.02 (0.64, 1.64) | 0.930 |
|  | Higher | 1.32 (0.80, 2.17) | 0.274 |
| Wealth index | Poorest | Reference | |
|  | Poorer | 1.07 (0.73, 1.56) | 0.732 |
|  | Middle | 1.25 (0.87, 1.78) | 0.225 |
|  | Richer | 1.38 (0.97, 1.96) | 0.073 |
|  | Richest | 1.82 (1.27, 2.61) | <0.01*** |
| Twin child | Single birth | Reference | |
|  | 1^st^ of multiple | 4.73 (2.41, 9.29) | <0.01*** |
|  | 2^nd^ of multiple | 5.31 (2.73, 10.32) | <0.01*** |
| Child is alive | No | Reference | |
|  | Yes | 0.39 (0.25, 0.62) | <0.01*** |
| Caesarean delivery | No | Reference | |
|  | Yes | 1.74 (1.36, 2.23) | <0.01*** |
| Size of child birth | Very large | Reference | |
|  | Larger than average | 0.30 (0.07, 1.36) | 0.120 |
|  | Average | 0.55 (0.15, 2.08) | 0.380 |
|  | Smaller than average | 6.79 (1.80, 25.59) | <0.01*** |
|  | Very small | 10.42 (2.29, 47.46) | <0.01*** |
|  | Don't know | 1 |  |
| During pregnancy Taking iron tablets/syrups | No | Reference |  |
|  | Yes | 1.64 (1.21, 2.21) | <0.01*** |
|  | Don't know | 1 |  |
| Birth interval | Very Short<(18m) | Reference | |
|  | Short (18-23m) | 0.32 (0.16, 0.66) | <0.01*** |
|  | Optimal (24-59m) | 0.53 (0.30, 0.95) | <0.05** |
|  | Long (≥60m) | 0.52 (0.30, 0.90) | <0.05** |
| ANC visits | <=2 Visit | Reference | |
|  | >2 Visit | 1.78 (1.39, 2.27) | <0.01*** |

**Note:** p-value<0.01, ‘***’, p<0.05, ‘**’
