# Supplementary material for: Exploring the multilevel determinants of low birth weight in Bangladesh: Understanding implications for targeted public health interventions
Source: PLOS Glob Public Health. 2026 Jan 23;6(1):e0005823. doi: 10.1371/journal.pgph.0005823 (PMC12829852; doi:10.1371/journal.pgph.0005823)
Supplement: Attachment — Submitted filename: Response_Reviewer.docx [file pgph.0005823.s004.docx]

**Author responses to the review comments:**

We would like to express our sincere gratitude to the reviewer and the Editor for their valuable comments. We have considered all the comments made by the reviewers and thoroughly revised and formatted the manuscript accordingly. A detailed response to each of the comments is provided in the table below:

| **First Reviewer comments:** | **Response** | **Note** |
| --- | --- | --- |
| General comments Repetition of text: In a few places (e.g., twin births as a predictor), the manuscript repeats point already made. You may wish to review the results section to streamline these and avoid redundancy. | We sincerely thank the reviewer for this helpful observation. We have carefully reviewed the Results section and removed repetitive statements, particularly regarding the role of twin births as a predictor of the outcome. Redundant explanations were consolidated to ensure concise and non-overlapping presentation of the findings. The revised Results section is now more streamlined and avoids unnecessary repetition. | Revised texts are in red colour. Please see the page number 5. |
| Specific comments: Background: Adding some text on how the inter-sectoral linkage between health, income and education are important will help lay a stronger foundation for the paper to be relevant. It will also support the choice of type of analysis. | We would like to thank the reviewer for this insightful suggestion. We have added a paragraph in the Background to highlight the inter-sectoral linkages between maternal education, household income, and access to healthcare in influencing low birth weight (LBW). | Revised texts are in red colour. Please see the page number 1. |
| Results section: The results are described in considerable detail. It might be helpful to focus on statistically significant predictors, while mentioning non-significant ones only when the adjusted analysis suggests a change. This would make the results section more concise and focused. | We appreciate the reviewer’s insightful suggestion. In the revised manuscript, we have focused on statistically significant predictors of low birth weight (LBW) in the results section. Non-significant variables are mentioned only when the adjusted analysis suggested notable changes in associations, ensuring that the results are concise, clear, and highlight the most relevant determinants. This revision enhances the readability and focusses of the results while maintaining scientific rigor | Revised texts are in red colour. Please see the page 7-12. |
| Descriptive table: The paper would benefit from a single, consolidated table describing the characteristics of the study population. This could replace the first table currently presented. It can also concise the results section as the first three tables essentially gives the same message. The unadjusted results can be described briefly with tables removed in annexes. The analysis has the opportunity to highlight on possible discrepancies in LBW across different groups and shed light on existing inequality | We sincerely thank the reviewer for this valuable suggestion. In response, we have consolidated the three tables into a single comprehensive table describing the characteristics of the study population, along with both unadjusted and adjusted results.   - The descriptive characteristics of the population are presented in one table for clarity. - Unadjusted results (simple odds ratios) are summarized in the same table, and full unadjusted tables are included in the annex. - Multilevel adjusted models are presented separately but in a concise format to highlight significant determinants of LBW. | Revised texts are in red colour. Please see the page 11 and 12. |
| Model selection: It would be useful to clarify whether stepwise inclusion methods (forward or backward elimination) were considered when identifying the best-fitting regression model. | Thank you so much for your wonderful comment. We have clarified the model selection procedure in the Methods section. Specifically, we applied a backward stepwise elimination approach based on the likelihood ratio test to identify the best-fitting logistic regression model. Variables that were significant in the bivariate analysis (p < 0.05) were initially included, and non-significant variables were sequentially removed until only those with p < 0.05 remained in the final model. | Revised texts are in red colour. Please see the page 6. |
| Conciseness of results: To reduce repetition, you might consider moving the first two or three detailed tables to the appendix as supplementary material, while presenting the key findings in a more concise form in the main text. | We would like to thank the reviewer to such a comment. We have already moved the first table as well as the Unadjusted OR logistic regression in the appendix section. We also concise the findings in the main text. | Revised texts are in red colour. Please see the page 7-16. |
| Data collection details: A short description of how LBW data were collected, along with any potential limitations (e.g., recall bias, measurement error), would add valuable context. | We appreciate the reviewer’s helpful suggestion. We have added a concise description of the data source and clarified how low birth weight (LBW) information was collected in the BDHS dataset, along with relevant limitations. | Revised texts are in red colour. Please see the page 5. |
| Discussion: The opening paragraph of the discussion currently overlaps with the background. It could instead be used to provide a clear summary of the main findings. | We appreciate the reviewer’s insightful comment. The opening paragraph of the Discussion has been revised to remove overlap with the Background section. It now provides a concise summary of the study’s main findings, thereby improving clarity and focus. | Revised texts are in red colour. Please see the page 17 and 18. |
| Expanding the discussion of how these results compare with or add to existing research could strengthen the manuscript’s contribution. Some of it has already been done but needs more. | Thank you for this valuable suggestion. We have expanded the Discussion to more clearly situate our findings within the existing literature. Additional comparisons with national and international studies have been incorporated to highlight how our results align with, differ from, or extend previous research. This enhancement strengthens the manuscript’s overall contribution and provides clearer context for the observed associations. | Revised texts are in red colour. Please see the page 18 and 19. |
| Discussion – limitations: Highlighting the key limitations of the analysis (data, methodology, generalisability) would provide a more balanced interpretation of findings. | We appreciate the reviewer’s suggestion. A dedicated limitations section has now been added to the Discussion. It highlights important data-related, methodological, and generalizability concerns to ensure a balanced interpretation of the findings. | Revised texts are in red colour. Please see the page 19. |
| Policy implications: It may be helpful to draw out the policy relevance of the findings—for example, how they could inform maternal and child health interventions to reduce LBW. | Thank you for this helpful suggestion. We have now added a paragraph outlining the policy implications of our findings, emphasizing how the identified determinants can inform maternal and child health interventions aimed at reducing low birth weight (LBW) in Bangladesh. | Revised texts are in red colour. Please see the page 19. |
| Evidence-based recommendations: Recommendations could be reinforced with references to existing research (e.g., evidence that increased antenatal care visits reduce LBW risk). | Thank you for the constructive suggestion. We have revised the Recommendations section to incorporate stronger evidence-based support, including references to studies demonstrating the effectiveness of antenatal care visits, maternal nutrition interventions, and community-based programs in reducing LBW risk. | Revised texts are in red colour. Please see the page 19 and 20. |
| Future research: A brief note on what additional research is needed would enhance the discussion. | Thank you for this insightful suggestion. We have added a brief statement outlining key areas for future research, including the need for longitudinal designs, additional contextual variables, and more advanced modelling approaches to strengthen understanding of the determinants of LBW. | Revised texts are in red colour. Please see the page 20. |
|  |  |  |

Finally, the revised manuscript has been produced following the valuable comments and suggestions of the reviewers. Once again, we would like to thank the reviewers for their sincere dedication, professional insights, and earnest cooperation in reviewing the manuscript.
